# The transfer of double morphologically good Day 5 blastocysts increases the risk of clinical pregnancy loss in singleton pregnancies following frozen-thawed embryo transfer

**DOI:** 10.3389/fendo.2025.1508014

**Published:** 2025-04-16

**Authors:** Yufeng Wang, Qin Wan, Xiaohui Lu, Lingjun Li, Huihui Wang, Li Chen, Xiuliang Dai

**Affiliations:** ^1^ The Center for Reproductive Medicine, Changzhou Maternal and Child Health Care Hospital, Changzhou Medical Center, Nanjing Medical University, Changzhou, Jiangsu, China; ^2^ Department of gynaecology and obstetrics, Xuancheng City Central Hospital, Xuancheng, Anhui, China; ^3^ Department of Obstetrics, Changzhou Maternal and Child Health Care Hospital, Changzhou Medical Center, Nanjing Medical University, Changzhou, Jiangsu, China

**Keywords:** single embryo transfer, double embryo transfer, clinical pregnancy loss, intrauterine inflammation, developmental defect

## Abstract

**Background:**

To investigate whether double embryo transfer (DET) increases the risk of spontaneous clinical pregnancy loss (CPL) in singleton pregnancies following frozen-thawed embryo transfer (FET), compared to single embryo transfer (SET).

**Methods:**

This retrospective cohort study included 2,448 females with singleton pregnancies (excluding vanishing twin cases) resulting from frozen-thawed single or double embryo transfers between January 2017 and September 2022. The CPL rate was the sole outcome measure. We compared CPL rates between SET and DET across three populations with increasing embryo developmental potential using binary logistic regression analysis: P1, comprising transfers of Day 3 cleavage-stage embryos; P2, comprising transfers of blastocysts; and P3, comprising transfers of top-quality blastocysts, defined as morphologically good Day 5 blastocysts.

**Results:**

After adjusting for confounding factors, the comparison between SET and DET revealed the following findings: in P1, DET had a slightly higher CPL rate compared to SET [OR (95% CI): 1.18 (0.74-1.90), p=0.46]; In P2, DET showed a moderately higher CPL rate [OR (95% CI): 1.34 (0.96-1.87), p=0.08]; In P3, DET had a significantly higher CPL rate [OR (95% CI): 1.55 (1.02-2.37), p=0.04]. A combined analysis indicated that as the developmental potential of the transferred embryo increased (from P1 to P2 and further to P3), the impact of DET on CPL also increased, as reflected by the rising OR values and decreasing p-values. We proposed that in singleton pregnancies resulting from DET, the loss of a non-viable embryo at a later stage, when it has a larger cell mass, may trigger excessive intrauterine inflammation, thereby increasing the risk of CPL for the remaining full developmental potential embryo. In singleton pregnancies resulting from DET, a higher-quality embryo that fails is more likely to die at a later stage. This could explain why the impact of DET on CPL increases with the developmental potential of the embryo.

**Conclusion:**

Since a significant difference in CPL between SET and DET was only observed in P3 population. Therefore, we concluded that compared to SET, the transfer of double morphologically good Day 5 blastocysts is associated with increased clinical pregnancy loss in singleton pregnancies following FET.

## Introduction

The number of embryos transferred is considered as a crucial factor influencing the clinical outcomes of *in vitro* fertilization-embryo transfer (IVF-ET). An increase in the number of embryos transferred has been found to significantly enhance the clinical pregnancy rate but also substantially increase the incidence of multiple pregnancies (1–4). Undoubtedly, multiple pregnancies significantly increase the risk of adverse gestational and perinatal outcomes, as well as short-term and long-term health complications for both mothers and babies (5–7). In natural pregnancies, the rate of multiple pregnancies is around 1%. By contrast, with assisted reproductive technology (ART) in China, the rate of multiple pregnancies exceeds 30% (8). To mitigate the high rate of multiple pregnancies, it is recommended that no more than two embryos be transferred per cycle in China. Consequently, most reproductive centers in China routinely transfer either one or two embryos per cycle.

Previous studies have reported a higher rate of adverse gestational and perinatal outcomes in singletons conceived by ART compared to those conceived spontaneously (9–12). The outcomes also differ between single embryo transfer (SET) and double embryo transfer (DET). Previous studies have indicated a higher risk of adverse outcomes in singletons from DET compared to SET, including the risk of neonatal death, low birthweight in frozen embryo transfer cycles, and very preterm birth and low birthweight in blastocyst transfer cycles (13–15). Theoretically, a singleton pregnancy from DET differs from a singleton pregnancy from SET. In a singleton pregnancy from DET, the surviving embryo could potentially be affected by the death of another embryo. It has been reported that embryonic apoptosis induces maternal sterile purulent inflammation, leading to the resorption of the dead embryo (16). It is possible that intrauterine inflammation resulting from the death of one embryo may adversely affect the surviving embryo, potentially leading to increased rates of adverse gestational and perinatal outcomes.

Here, we investigated whether singleton pregnancies from DET have a higher likelihood of spontaneous clinical pregnancy loss (CPL) compared to SET. A previous study has suggested a higher rate of missed abortion in patients with singleton pregnancies conceived after multiple embryo transfers (17). Nonetheless, the small sample size (only 195 singleton pregnancies) limits the conclusiveness of this finding. In the present study, we retrospectively analyzed frozen-thawed embryo transfer (FET) cycles with singleton pregnancies at our reproductive center from January 2017 to December 2022.

## Materials and methods

### Study design

**Figure 1:**
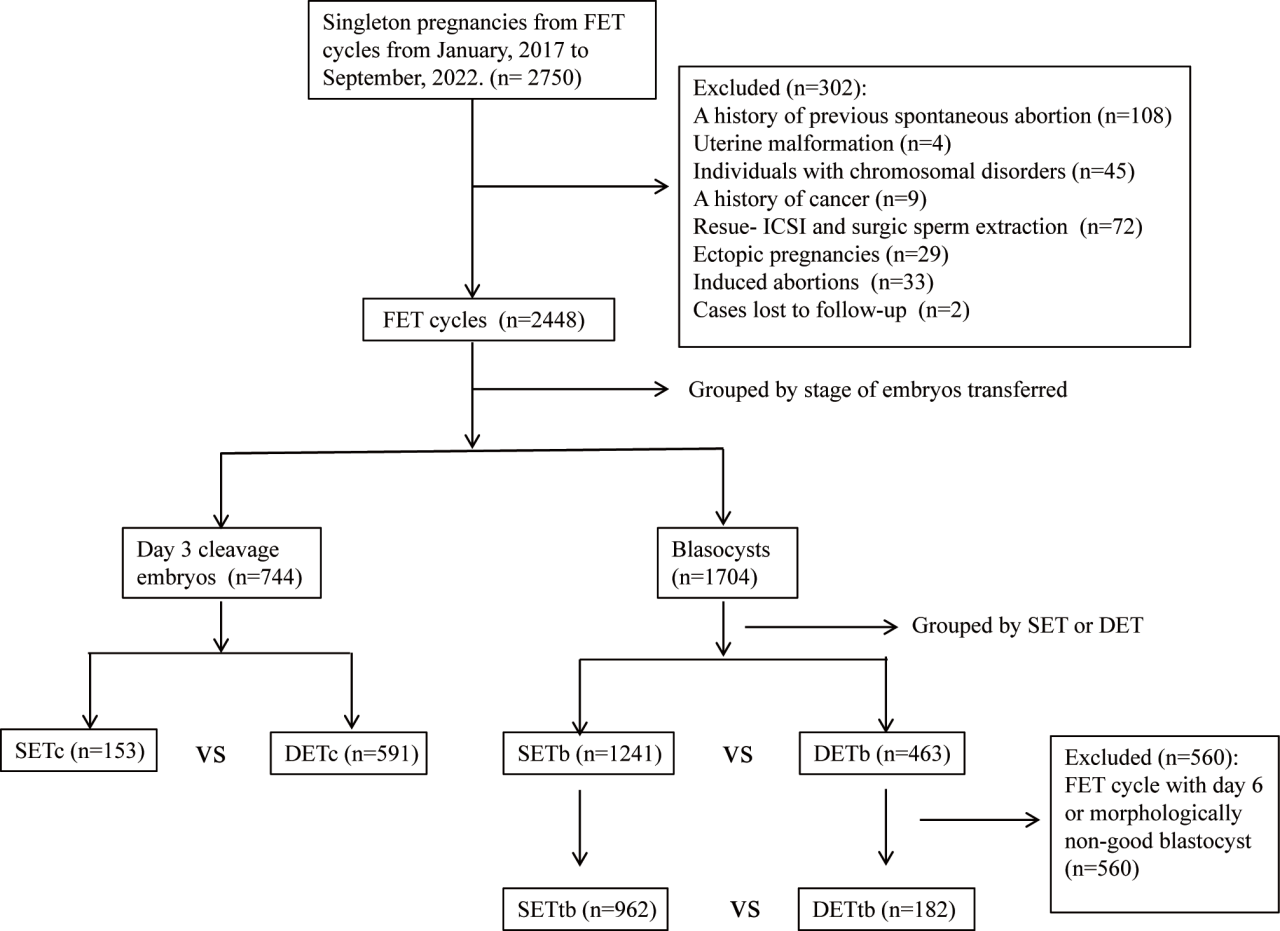
Study flow diagram. SET, single embryo transfer; DET, double embryo transfer; SETc, SET with Day 3 cleavage embryo; DETc, DET with Day 3 cleavage embryo; SETb, SET with blastocyst; DETb, DET with blastocyst; SETtb, SET with top blastocyst; DETtb, DET with top blastocyst.

This was a retrospective study. This study collected data from couples with confirmed singleton pregnancies via FET at our reproductive center from January 2017 to September 2022. This study was approved by the ethics committee of Changzhou Maternal and Child Health Care Hospital. Given the difference in embryo stage, the transfer cycles were initially divided into two groups: P1(Day 3 cleavage embryos) and P2 (blastocysts). Subsequently, each group was further subdivided based on the number of embryos transferred into SET and DET groups. In addition, data from SET and DET with top blastocyst (morphologically good Day 5 blastocysts, P3 group) were extracted for analysis. Baseline characteristics and the rate of CPL between SET and DET were compared in P1 and P2, respectively. Binary logistic regression analysis was used to evaluate the effect of DET on the occurrence of CPL in P1, P2 and P3, respectively. Further details of the study design were described in **Figure 1**.

### Inclusion/exclusion criteria

FET cycles with a confirmation of singleton pregnancies (one heartbeat confirmed by the first ultrasonography) were included;

Females with a history of previous spontaneous abortion or uterine malformation were excluded;

Individuals with chromosomal disorders or a history of cancer were excluded;

Surgical sperm extraction and rescue-ICSI were excluded;

FET cycles resulting in ectopic pregnancies or induced abortions were excluded;

Cases lost to follow-up were excluded.

### Embryo culture procedures

Females underwent controlled stimulation protocols to promote follicle growth. Oocytes were collected approximately 36 hours after the trigger under ultrasound guidance, and fertilization was performed with either conventional IVF or the ICSI method. Zygotes were evaluated on day 1, and embryos were morphologically scored on Day 3, 5, or 6. Day 3 cleavage embryos were frozen on Day 3, while blastocysts were frozen on Day 5 or 6.

### Morphological score of embryos

The morphological scoring for Day 3 cleavage embryos and blastocysts was performed as previously described (18). Briefly, Day 3 embryos were classified into four grades based on their morphological appearance. Grades I and II were considered good embryos, grade III as non-good embryo, and grade IV embryos were discarded. For blastocysts, a morphologically good blastocyst was defined as having a blastocyst expansion grade over 3, with an A or B score for both the inner cell mass (ICM) and trophectoderm (TE). A morphologically non-good blastocyst was defined as having a blastocyst expansion grade over 3, a C score for either the ICM or TE, and an A or B score for the corresponding opposite component (TE or ICM).

### Definitions

*A singleton pregnancy* is defined as a pregnancy where only one fetus is present, confirmed by the detection of a single heartbeat on the first ultrasonography.

*Top blastocysts* refer to morphologically good blastocysts that were formed on Day 5, characterized by a high degree of blastocyst expansion (over 3) and an A or B score for both the ICM and TE.

*Clinical pregnancy loss* refers to the spontaneous loss of a clinical pregnancy.

### Embryo culture and transfer strategy

In late 2016, our reproductive center implemented blastocyst culture. To minimize the risk of having no embryos available for transfer, it’s a common practice not to perform blastocyst culture for couples with a very limited number of Day 3 cleavage embryos (<=4). In the case of a moderate number of Day 3 cleavage embryos (>4 and <=7), the two to four best Day 3 embryos are frozen, while the remaining embryos undergo further culture. When the number of Day 3 cleavage embryos is =>7 and the number of Grade I and II embryos is =>4, all embryos are subjected to further culture. For embryo transfer priority, the order is as follows: morphologically good Day 5 blastocysts have the highest priority, followed by morphologically good blastocysts, then good Day 3 embryos, and finally non-optimal blastocysts.

### Statistical analysis

Data were analyzed using SPSS software (Version 21, IBM). Continuous data were first examined by the Normality and Lognormality test. Data that were not normally distributed were compared using the Mann–Whitney U test. The constituent data were compared using the chi-square test. Binary logistic regression analysis was used to evaluate the impact of groups (DET vs. SET) on the occurrence of CPL, while adjusting for potential confounding factors including female age and BMI, semen DFI, number of oocytes retrieved, type of infertility, infertility duration, infertility cause, previous transfer cycle, endometrium preparation, and embryo quality. *p*< 0.05 was considered statistically significant.

## Results

### Characteristics of patients, ovarian stimulation, embryo transfer, and the occurrence of clinical pregnancy loss

In Day 3 cleavage embryo transfer cycles (P1), both the SET and DET groups displayed numerous similar baseline characteristics, including infertility duration, primary diagnoses, as well as BMI of both females and males, females and males with age over 35 years, semen DFI, males with semen DFI over 30, previously failed transfer cycles, ovarian stimulation protocols, fertilization methods, endometrial preparation protocols, endometrial thickness, and the proportion of transferred grade III embryos (**Table 1**). However, compared to the SET group, the DET group had a higher proportion of patients with primary infertility, younger ages for both females and males at oocyte retrieval or embryo transfer, a higher proportion of patients with at least one previous IVF cycle, a higher number of oocytes retrieved, and a higher proportion of transfer cycles with at least one grade III embryo (**Table 1**). The CPL rate was similar between the SET and DET groups (**Table 1**).

**Table 1:** Description of cohort.

Characteristics	Day 3 cleavage embryo			Blastocyst		
	SETc	DETc	*p*	SETb	DETb	*p*
FET cycles	153	591		1241	463	
Primary infertility (%)	68 (44.4)	357 (60.4)	0	701 (56.5)	259 (55.9)	0.84
Infertility duration	3 [1.5, 5]	3 [2,4]	0.77	3 [2, 4]	3 [2, 4]	0.55
Primary diagnosis (%)						
Tubal factor	59 (38.6)	263 (44.5)		620 (50.0)	229 (49.5)	
DOR	38 (24.8)	103 (17.4)	0.23	40 (3.2)	13 (2.8)	0.95
Ovulatory dysfunction	13 (8.5)	39 (6.6)		178 (14.3)	73 (15.8)	
Male factor	18 (11.8)	79 (13.4)		171 (13.8)	64 (13.8)	
Others	25 (16.3)	107 (18.1)		232 (18.7)	84 (18.1)	
Female Age						
at oocyte retrieval	32 [29, 35]	31 [28, 35]	0.04	30 [28, 33]	29 [27, 32]	0.01
>35 years old	36 (23.5)	127 (21.5)	0.58	133 (10.7)	46 (9.9)	0.64
at embryo transfer	33 [30, 36]	31 [29, 35]	0.04	31 [28, 33]	30 [28,33]	0.01
>35years old	41 (26.8)	137 (23.2)	0.35	154 (12.4)	52 (11.2)	0.51
Male age						
at oocyte retrieval	33 [29, 37]	31 [29,35]	0.03	31 [29, 34]	30 [28,33]	0
at embryo transfer	33 [30, 37]	32 [29, 36]	0.02	32 [29, 35]	31 [29,34]	0
BMI						
Female	22.3 [20.13, 25.3]	22 [20, 24.4]	0.39	22 [19.9, 25.0]	21.8[19.9, 24.8]	0.47
>30	12 (7.8)	20 (3.4)	0.02	65 (5.2)	13 (2.8)	0.03
Male	24.6 [22.6,27.7]	24.4 [22.4,27]	0.21	24.6 [22.4, 27.1]	24.3 [22.3, 27.2]	0.36
Semen DFI	14.0 [8.73,21.84]	12.7 [8.27, 20.19]	0.33	12.8 [8.2, 19.1]	11.1 [7.1,17.7]	0.01
>30	9 (5.88)	27 (4.57)	0.5	79 (6.4)	25 (5.4)	0.46
Previous IVF cycles (%)						
0	119 (77.78)	443 (74.96)		1134 (91.4)	420 (90.7)	
1	11 (7.19)	100 (16.92)	0.01	92 (7.4)	38 (8.2)	0.84
=>2	23 (15.03)	48 (8.12)		15 (1.2)	5 (1.1)	
Previous transfer cycles (%)						
0	102 (66.7)	389 (65.8)		937 (75.5)	302 (65.2)	
1	36 (23.5)	141 (23.9)	0.99	222 (17.9)	112 (24.2)	0
=>2	15 (9.8)	56 (10.3)		82 (6.6)	49 (10.6)	
GnRH analogues						
Agonist	56 (63.6)	264 (44.7)		906 (73.0)	363 (78.4)	
Antagonist	26 (17.0)	106 (17.9)	0.11	274 (22.1)	83 (17.9)	0.07
No analogues	71 (46.4)	221 (37.4)		61 (4.9)	17 (3.7)	
Oocyte retrieved	5 [3, 10]	7 [5, 11]	0	13 [10, 17]	13 [11-17]	0.11
Fertilization methods						
IVF	120 (78.4)	473 (80.0)	0.66	1060 (85.4)	411 (88.8)	0.07
ICSI	33 (21.6)	118 (20.0)	0.66	181 (14.6)	52 (11.2)	0.07
Endometrium preparation						
Artificial cycle	114 (74.5)	436 (73.8)		997 (80.3)	342 (73.9)	
Ovarian stimulation cycle	29 (19.0)	122 (20.6)	0.83	208 (16.8)	107 (23.1)	0.01
Modified natural cycle	10 (6.5)	33 (5.6)		36 (2.9)	14 (3.0)	
Thickness of endometrium	9.75 [8.63, 11.0]	9.3 [8.1, 10.7]	0.12	9 [8, 10]	9 [8, 10]	0.83
Embryo quality (%)						
I/II	144 (94.1)	1061 (89.8)	0.09	/	/	
Cycle with at least one grade III embryo	9 (5.9)	112 (19.0)	0	/	/	
Morphologically good blastocyst	/	/		1204 (97.0)	753 (81.3)	0
Cycle with at least one morphologically non-good blastocyst	/	/		37 (3.0)	148 (32.0)	0
Day 5 blastocyst	/	/		936 (75.4)	508 (54.9)	0
Cycles with at least one day 6 blastocyst	/	/		268 (21.6)	252 (54.4)	0
CPL (%)	30 (19.1)	127 (21.5)	0.61	200 (16.1)	93 (20.1)	0.05

FET, frozen-thawed embryo transfer; declined ovarian reserve; DFI, DNA fragmentation index; CPL, clinical pregnancy loss.

Data are presented as the median [the first quartile, the third quartile] or count (percentage).

In blastocyst transfer cycles (P2), both the SET and DET groups displayed numerous similar baseline characteristics, including primary infertility, infertility duration, primary diagnoses, females and males with age over 35 years, BMI of both males and females, males with semen DFI over 30, previous IVF cycles, ovarian stimulation protocols, oocytes retrieved, fertilization method, and endometrial thickness (**Table 1**). However, compared to the SET group, the DET group had younger ages for both females and males at oocyte retrieval or embryo transfer, less females with BMI over 30, lower semen DFI, more patients with at least a previous failed transfer cycles, a higher percentage of endometrial preparation with ovarian stimulation protocol, fewer Day 5 or morphologically good blastocysts transferred, and more transfer cycles with at least one day 6 embryo or one morphologically non-good blastocyst (**Table 1**). The CPL rate was higher in the DET group than in the SET group, although the difference was not significant (**Table 1**).

### The effect of DET on the occurrence of CPL among groups

Here, we evaluated the effect of DET on the occurrence of CPL in three distinct groups, P1, P2, and P3. The developmental potential of embryos in these three groups gradually increased from P1 (Day 3 cleavage embryos) to P2 (blastocysts) and to P3 (top blastocysts).Variables including the number of oocytes retrieved, female age (at oocyte retrieval), infertility duration, infertility cause, type of infertility, female BMI, semen DFI, previous transfer cycle, protocols of endometrial preparation, and embryo quality were considered as confounding factors. After adjusting for confounding factors, compared to SET, DET showed a slightly higher rate of CPL in P1[OR (95% CI): 1.18 (0.74-1.90), p=0.46] (**Table 2**); a moderate higher rate of CPL in P2 [1.34 (0.96-1.87), p=0.08] (**Table 3**); a significant higher rate of CPL in P3 [1.55 (1.02-2.37), p=0.04] (**Table 4**).

**Table 2:** The effect of DET on the occurrence of CPL for Day 3 cleavage embryo transfer.

	OR (95% CL)	*p*
Occytes retrieved (n=744)	1.00 (0.95-1.05)	0.97
Female age (n=744)	1.12 (1.07-1.18)	0.00
Type of infertility		
Primary infertility (n=429)	1 (ref)	
Secondary infertility (n=315)	0.84 (0.56-1.23)	0.39
Infertility duration (n=744)	0.96 (0.89-1.04)	0.31
Infertility causes		
Male factor (n=97)	1 (ref)	
Ovarian function decline (n=141)	0.76 (0.38-1.53)	0.45
*Others (n=506)	0.62 (0.36-1.08)	0.09
BMI		
<=30 (n=712)	1 (ref)	
>30 (n=32)	0.64 (0.26-1.54)	0.49
DFI		
<=30 (n=708)	1 (ref)	
>30 (n=36)	0.50 (0.19-1.35)	0.17
Previous transfer cycle		
0 (n=497)	1 (ref)	
1 (n=177)	1.17 (0.75-1.83)	0.50
>1 (n=70)	1.11 (0.59-2.10)	0.74
Endometrium preparation		
Ovarian stimulation cycle (n=148)	1 (ref)	
Artificial cycle (n=552)	0.76 (0.46-1.24)	0.27
Modified natural cycle (n=44)	0.96 (0.44-2.11)	0.92
Embryo quality		
Good embryos (n=624)	1 (ref)	
Containing at least one grade III embryo (n=120)	1.13 (0.68-1.86)	0.64
Group		
SET (n=153)	1 (ref)	
DET (n=591)	1.18 (0.74-1.90)	0.46

Data are presented as the OR (95% CL).*Others includes tubal factor, ovulatory dysfunction and other factor. DFI, DNA fragmentation index; BMI, body mass index; SET, single embryo transfer; DET, double embryo transfer.

**Table 3:** The effect of DET on the occurrence of CPL for blastocyst transfer.

	OR (95% CL)	*p*
Occytes retrieved (n=1704)	0.99 (0.96-1.01)	0.36
Female age (n=1704)	1.06(1.00-1.10)	0.00
Type of infertility		
Primary infertility (n=960)	1 (ref)	
Secondary infertility (n=744)	1.00 (0.76-1.32)	0.99
Infertility duration (n=1704)	1.00 (0.94-1.07)	0.93
Infertility causes		
Male factor (235)	1 (ref)	
Declined ovarian reserve (53)	1.06 (0.50-2.27)	0.88
*Others (1416)	1.02 (0.69-1.51)	0.92
BMI		
<=30 (n=1626)	1 (ref)	
>30 (n=78)	1.56 (0.90-2.70)	0.12
DFI		
<=30 (n=1600)	1 (ref)	
>30 (n=104)	1.03 (0.60-1.79)	0.91
Previous transfer cycle		
0 (n=1239)	1 (ref)	
1 (n=334)	0.74 (0.51-1.06)	0.10
>1 (n=131)	1.45 (0.93-2.25)	0.10
Endometrium preparation		
Ovarian stimulation cycle (n=315)	1 (ref)	
Artificial cycle (n=1339)	1.27 (0.88-1.82)	0.20
Modified natural cycle (n=50)	0.98 (0.42-2.26)	0.96
Embryo morphology		
Good (n=1519)	1 (ref)	
One non-good embryo (n=122)	1.23 (0.72-2.11)	0.44
Non-good (n=63)	1.97 (1.13-3.50)	0.02
Days of embryo		
Day 5 (n=1183)	1 (ref)	
Day 5+ day 6 (n=86)	0.55 (0.27-1.13)	0.10
Day 6 (n=435)	1.39 (1.03-1.89)	0.03
Group		
SET (n=1241)	1 (ref)	
DET (n=463)	1.34 (0.96-1.87)	0.08

Data are presented as the OR (95% CL).*Others includes tubal factor, ovulatory dysfunction and other factor. DFI, DNA fragmentation index; BMI,: body mass index; SET, single embryo transfer; DET, double embryo transfer.

**Table 4:** The effect of DET on the occurrence of CPL for top blastocyst transfer.

	OR (95% CL)	p
Occytes retrieved (n=1144)	0.98 (0.95-1.01)	0.15
Female age (n=1144)	1.05 (1.00-1.11)	0.04
Type of infertility		
Primary infertility (n=635)	1 (ref)	
Secondary infertility (n=509)	1.09 (0.76-1.55)	0.65
Infertility duration (n=1144)	1.01 (0.93-1.09)	0.85
Infertility causes		
Male factor (150)	1 (ref)	
Declined ovarian reserve (34)	1.04 (0.93-2.80)	0.94
*Others (960)	0.85 (0.52-1.40)	0.53
BMI		
<=30 (n=1091)	1 (ref)	
>30 (n=53)	0.91 (0.40-2.08)	0.83
DFI		
<=30 (n=1078)	1 (ref)	
>30 (n=66)	1.36 (0.70-2.67)	0.37
Previous transfer cycle		
0 (n=907)	1 (ref)	
1 (n=182)	0.65 (0.39-1.10)	0.11
>1 (n=55)	1.11 (0.52-2.34)	0.80
Endometrium preparation		
Ovarian stimulation cycle (n=203)	1 (ref)	
Artificial cycle (n=901)	1.64 (0.99-2.72)	0.05
Modified natural cycle (n=30)	0.79 (0.22-2.85)	0.71
Group		
SET (n=962)	1 (ref)	
DET (n=182)	1.55 (1.02-2.37)	0.04

Data are presented as the OR (95% CL).*Others includes tubal factor, ovulatory dysfunction and other factor. DFI, DNA fragmentation index; BMI, body mass index; SET, single embryo transfer; DET, double embryo transfer.

### Proposed mechanism

**Figure 2:**
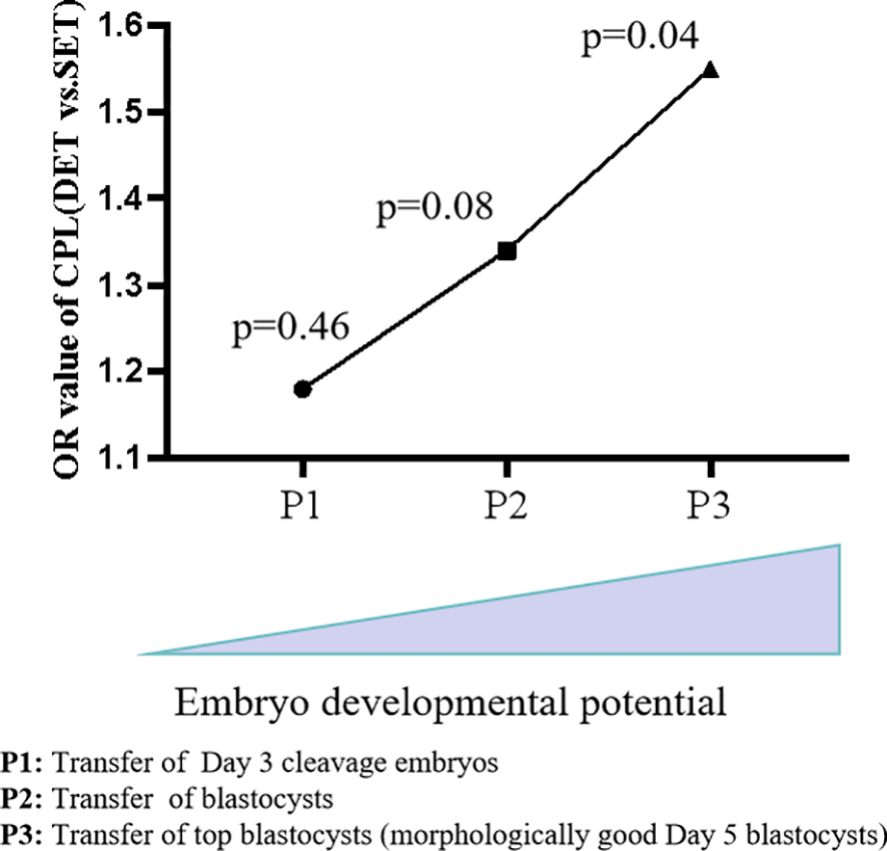
Combined analysis of DET on the occurrence of CPL across groups. As the potential of transferred embryos increases (from Day 3 cleavage embryos to blastocysts and further to top blastocysts), the impact of DET on the occurrence of CPL became progressively evident. CPL, Clinical pregnancy loss; SET, single embryo transfer; DET, double embryo transfer.

**Figure 3:**
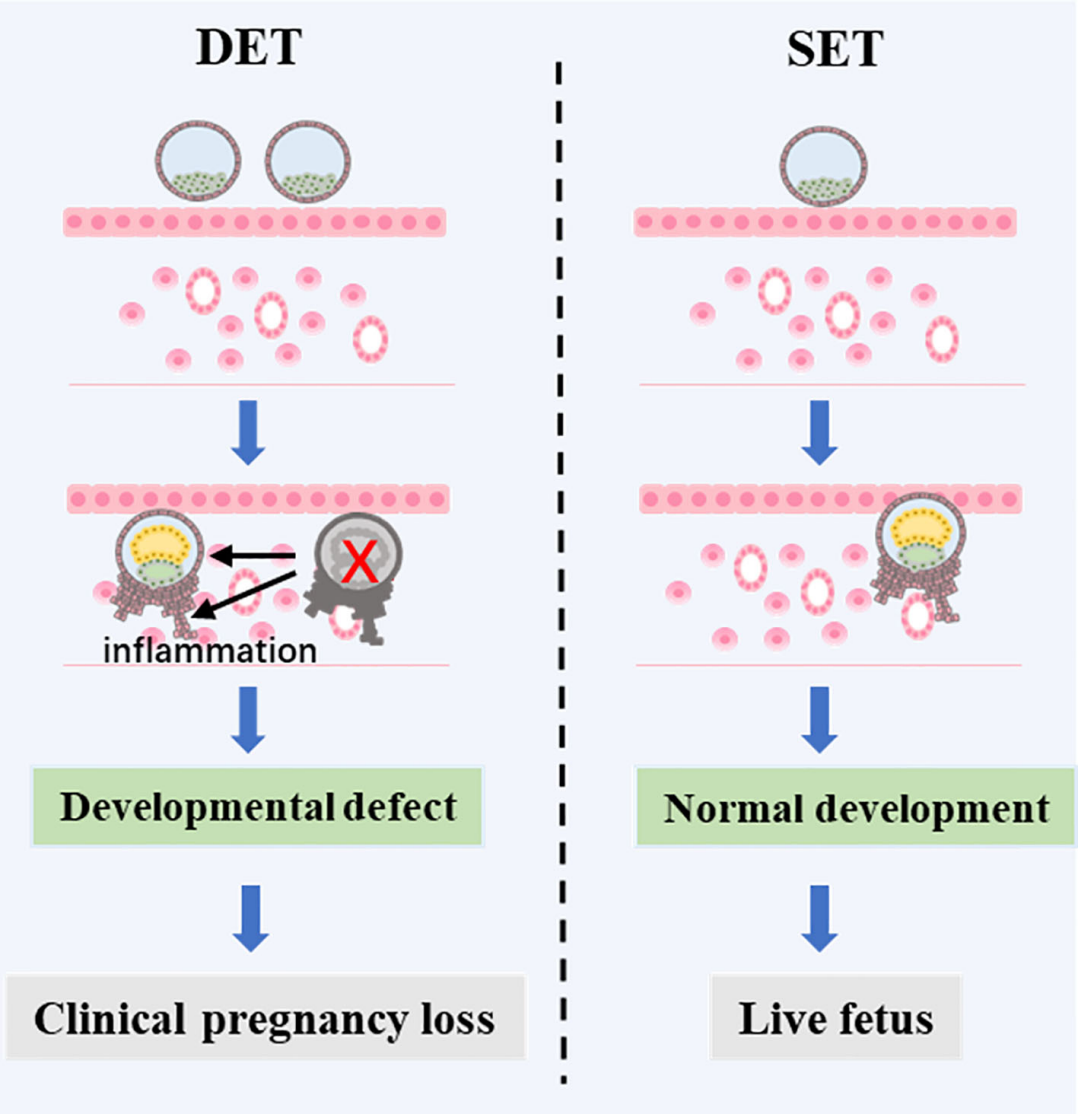
A proposed mechanism of CPL caused by DET. In DET, two embryos are transferred, one with full developmental potential and one with limited developmental potential. The embryo with limited potential may establish a biochemical pregnancy but then fail to progress to a clinical pregnancy. This failure triggers an inflammatory response in the uterus, which negatively affects the development of the surviving embryo with full developmental potential, ultimately leading to the loss of the clinical pregnancy. However, if the embryo with full developmental potential were transferred alone, a live birth could be achieved.

A combined analysis showed that DET progressively increases the risk of CPL as the developmental potential of the embryos rises (from P1 to P2, and further to P3) (**Figure 2**). We proposed that in singleton pregnancies following DET, the loss of one embryo at a later developmental stage (potentially after biochemical pregnancy) may trigger excessive intrauterine inflammation, leading to the loss of the clinically established pregnancy of the remaining full potential embryo (**Figure 3**). In singleton pregnancies after DET, as the developmental potential of the transferred embryos increases, the likelihood of later-stage embryo loss also rises, which explains this observation. Due to being unaffected by the loss of another embryo, an embryo with full developmental potential may result in a live birth using the SET strategy (**Figure 3**).

## Discussion

In the present study, we investigated whether DET increases the risk of CPL in singleton pregnancies, compared to SET. Previous studies have shown adverse outcomes in singleton births following DET compared to SET, suggesting that the death of one embryo indeed influences the remaining surviving embryo with full potential (13, 19, 20). In the present study, we found that as the potential of transferred embryos increased (from Day 3 cleavage embryos to blastocysts to top blastocysts), the effect of DET on the occurrence of CPL became progressively more evident in singleton pregnancies. More importantly, we found that transferring two morphologially good Day 5 blastocyst significantly increases the CPL rate in singleton pregnancies following FET, compared to the transfer of single morphologially good Day 5 blastocyst.

For Day 3 cleavage embryo transfer, it was common practice to transfer two embryos (if available). Owing to the higher developmental potential of blastocyst, SET was more commonly used for blastocyst transfer, particularly for morphologically good Day 5 blastocysts. Consequently, in the Day 3 cleavage embryo transfer group, most singleton pregnancies were achieved thorough DET, while in the blastocyst transfer group, most singleton pregnancies were achieved through SET. Our embryo culture and transfer strategy, as outlined in the Methods section, may contribute to the older age and lower ovarian reserve in couples receiving SET in the present study. For DET, transferring one good embryo and one non-good embryo is more likely to result in a singleton pregnancy, compared to transferring two good or two non-good embryos. The morphological score of the blastocyst also determines whether one or two embryos are transferred. Therefore, the embryo quality in the DET group was lower compared to SET in the present study. Additionally, a failed transfer cycle with a good embryo often leads to the decision to transfer two embryos in the subsequent cycle. Consequently, the DET group had more couples with at least one failed transfer cycle, particularly in blastocyst transfer. The heterogeneity between the SET and DET populations could potentially introduce bias into the entire study. However, variables such as age, embryo quality, infertility duration and previous transfer cycles were considered as confounding factors in the analysis, which help to mitigate potential bias.

In natural conception, the zygote develops into a Day 3 cleavage embryo and progresses further into a blastocyst within the oviduct (21–23). Then, the blastocyst moves into the uterine cavity, hatches from the zona pellucida, and initiates the process of implantation (21). The interaction between the blastocyst and the endometrium, along with the rapid growth of the blastocyst, stimulates the secretion of β-HCG, leading to a biochemical pregnancy (24, 25). Following this, the embryo continues to grow, developing a fetalheart, which leads to a clinical pregnancy (26). In ART, the processes that occur in the oviduct are replicated *in vitro*, within a laboratory dish (27). In humans, embryonic loss is common and can occur at any stage of development, including before or at the blastocyst stage, after a biochemical pregnancy (biochemical pregnancy loss), or after the establishment of clinical pregnancy (clinical pregnancy loss) (28–30).

It has been reported that the embryonic apoptosis can directly trigger maternal sterile purulent inflammation, leading to the resorption of the dead embryo (16). It is well established that some degree of systemic or uterine inflammation is necessary both for normal implantation and pregnancy while excessive inflammation can increase the risk of miscarriage (31). Evidence from ART indicates that higher levels of serum C-reactive protein, which reflects inflammation, are associated with pregnancy loss (32). A recent prospective study showed a significantly higher of pro-inflammatory cytokines at day 16 following embryo transfer (during biochemical pregnancy, but before clinical pregnancy) in women who experienced subsequent miscarriage (33), indicating that an excess of pro-inflammatory factors during implantation increases the risk of CPL. Based on these studies, it is reasonable to assume that in singleton pregnancies following DET, the death of an embryo at a later developmental stage, when it has a larger cell mass, may trigger an excess inflammatory response, significantly increasing the risk of loss of the remaining full potential embryo. It is well established that embryo developmental potential is tightly associated with clinical outcomes. There should be an order of embryo developmental potential, live birth embryos > clinical pregnancy embryos > biochemical pregnancy embryos > blastocysts > Day3 cleavage embryos. In the setting of singleton pregnancies following DET, the likelihood of the decreased embryo dying at later stage should follow this order, morphologically good Day 5 embryos > blastocysts > Day3 cleavage embryos. This well explain the observation in the present study that, as the developmental potential of transferred embryos increases, the effect of DET on the occurrence of CPL becomes more pronounced.

Our findings are supported by several studies. A recent study analyzing data from 4232 women showed that DET had a lower cumulative live birth rate compared to SET [OR (95% CL): 0.76 (0.53–1.07)] (34). Another study, including data from 49,333 patients, demonstrated that iSET is associated with a significantly higher cumulative livebirth rate [OR (95% CL): 1.32 (1.26 –1.38)], compared to iDET (35). Clua et al. analyzing data from 1139 oocyte donation cycles, revealed that the cumulative pregnancy and livebirth rates of SET vs. DET is 82.8% vs. 77.2% and 76.4% vs. 63.7% respectively (36). This indicates a higher rate of CPL in the DET group (SET vs DET: 6.4% vs. 12.7%). A more recent study has shown that DET increases the rate of CPL for females receiving euploid frozen blastocyst transfer compared to SET (37). More importantly, the results from these studies can be well explained by the present study, which suggests that the DET strategy may result in the loss of full-potential embryos in singleton pregnancies.

The findings of this study are clinically significant. While DET significantly increases the risk of twin pregnancies, it remains prevalent in reproductive centers. One key reason for this is that many patients consider twin pregnancies acceptable. However, this study revealed that transferring two morphologically good Day 5 blastocysts increased the risk of CPL in singleton pregnancies, a risk that would be unacceptable to most patients. These findings may encourage the broader adoption of the SET strategy. A recently published committee opinion on embryo transfer limits suggests that for women aged 38 years or older, transferring two or more embryos may be considered (38). Full-potential embryos are particularly valuable for patients with poor prognoses. Therefore, based on our findings, SET should be prioritized to avoid the possible loss of full potential embryos. Additionally, our study provides novel evidence that supports the most recent ESHRE guidelines on the number of embryos to transfer during IVF/ICSI, which emphasize that no clinical or embryological factors alone justify recommending DET over eSET (39).

The present study has several strengths. Firstly, its design is robust, as we analyzed the impact of DET on the occurrence of CPL in singleton pregnancies across three groups: the Day 3 cleavage embryo group, the blastocyst group, and the top blastocyst group, each representing progressively increased potential of the dead embryo. Secondly, we proposed a theory that helps explain our observations effectively. Thirdly, the primary findings of our study are supported by a large cohort study involving 49,333 patients (35). However, owing to the retrospective nature of the study, there is a possibility that important confounding factors were not fully accounted for. Moreover, the sample size of our study may have been insufficient to achieve statistical significance for certain comparisons, such as transfer of Day 3 cleavage embryos. Additionally, the data utilized in the present study were derived from a single reproductive center. We encourage reproductive centers, particularly large ones, to repeat our analysis using their own data to test our findings. Furthermore, since our study was based on data from FET cycles, the applicability of our conclusions to fresh embryo transfer cycles warrants further investigation.

## Conclusion

Since a significant difference in CPL between SET and DET was only observed in the population with the transfer of morphologically good Day 5 blastocysts, we concluded that, compared to SET, the transfer of double morphologically good Day 5 blastocysts is associated with an increased risk of clinical pregnancy loss in singleton pregnancies following FET. Whether double transfers of Day 3 cleavage embryos or non-morphologically good Day 5 blastocysts increase the risk of CPL in singleton pregnancies, compared to single transfers of Day 3 cleavage embryos or non-morphologically good Day 5 blastocysts, still needs to be tested with large datasets.

## Data Availability

The original contributions presented in the study are included in the article/supplementary material. Further inquiries can be directed to the corresponding authors.

## References

[1] Kerin JF Warnes GM Quinn PJ Jeffrey R Kirby C Matthews CD. Incidence of multiple pregnancy after *in-vitro* fertilisation and embryo transfer. Lancet. (1983) 2:537–40. doi: 10.1016/S0140-6736(83)90569-X 6136693

[2] Komori S Kasumi H Horiuchi I Hamada Y Suzuki C Shigeta M. Prevention of multiple pregnancies by restricting the number of transferred embryos: randomized control study. Arch Gynecol Obstet. (2004) 270:91–3. doi: 10.1007/s00404-003-0513-x 12908110

[3] Bronson R. How should the number of embryos transferred to the uterus following *in-vitro* fertilization be determined to avoid the risk of multiple gestation? Hum Reprod. (1997) 12:1605–7. doi: 10.1093/humrep/12.8.1605 9308775

[4] Gelbaya TA Tsoumpou I Nardo LG. The likelihood of live birth and multiple birth after single versus double embryo transfer at the cleavage stage: a systematic review and meta-analysis. Fertil Steril. (2010) 94:936–45. doi: 10.1016/j.fertnstert.2009.04.003 19446809

[5] Qin J Wang H Sheng X Liang D Tan H Xia J. Pregnancy-related complications and adverse pregnancy outcomes in multiple pregnancies resulting from assisted reproductive technology: a meta-analysis of cohort studies. Fertil Steril. (2015) 103:1492–508 e1-7. doi: 10.1016/j.fertnstert.2015.03.018 25910567

[6] Wei J Wu QJ Zhang TN Shen ZQ Liu H Zheng DM. Complications in multiple gestation pregnancy: A cross-sectional study of ten maternal-fetal medicine centers in China. Oncotarget. (2016) 7:30797–803. doi: 10.18632/oncotarget.v7i21 PMC5058718 27127170

[7] Doyle P. The outcome of multiple pregnancy. Hum Reprod. (1996) 11 Suppl 4:110–7; discussion 118-20. doi: 10.1093/humrep/11.suppl_4.110 9147114

[8] Hu L Bu Z Huang G Sun H Deng C Sun Y. Assisted reproductive technology in China: results generated from data reporting system by CSRM from 2013 to 2016. Front Endocrinol (Lausanne). (2020) 11:458. doi: 10.3389/fendo.2020.00458 33042000 PMC7527788

[9] Pinborg A Wennerholm UB Romundstad LB Loft A Aittomaki K Soderstrom-Anttila V. Why do singletons conceived after assisted reproduction technology have adverse perinatal outcome? Systematic review and meta-analysis. Hum Reprod Update. (2013) 19:87–104. doi: 10.1093/humupd/dms044 23154145

[10] Wong K Carson KR Crane J. Risk of stillbirth in singleton gestations following *in vitro* methods of conception: a systematic review and meta-analysis. BJOG. (2021) 128:1563–72. doi: 10.1111/1471-0528.16691 33683788

[11] Stojnic J Radunovic N Jeremic K Kotlica BK Mitrovic M Tulic I. Perinatal outcome of singleton pregnancies following *in vitro* fertilization. Clin Exp Obstet Gynecol. (2013) 40:277–83. 23971259

[12] Sazonova A Kallen K Thurin-Kjellberg A Wennerholm UB Bergh C. Obstetric outcome after *in vitro* fertilization with single or double embryo transfer. Hum Reprod. (2011) 26:442–50. doi: 10.1093/humrep/deq325 21126967

[13] Rodriguez-Wallberg KA Palomares AR Nilsson HP Oberg AS Lundberg F. Obstetric and perinatal outcomes of singleton births following single- vs double-embryo transfer in Sweden. JAMA Pediatr. (2023) 177:149–59. doi: 10.1001/jamapediatrics.2022.4787 PMC9857532 36469325

[14] Martin AS Chang J Zhang Y Kawwass JF Boulet SL McKane P. Perinatal outcomes among singletons after assisted reproductive technology with single-embryo or double-embryo transfer versus no assisted reproductive technology. Fertil Steril. (2017) 107:954–60. doi: 10.1016/j.fertnstert.2017.01.024 PMC11350526 28292615

[15] Wu Y Chen W Zhou L Gao X Xi X. Single embryo transfer improve the perinatal outcome in singleton pregnancy. J Matern Fetal Neonatal Med. (2020) 33:3266–71. doi: 10.1080/14767058.2019.1571029 30646782

[16] Drews B Landaverde LF Kuhl A Drews U. Spontaneous embryo resorption in the mouse is triggered by embryonic apoptosis followed by rapid removal via maternal sterile purulent inflammation. BMC Dev Biol. (2020) 20:1. doi: 10.1186/s12861-019-0201-0 31918653 PMC6953269

[17] Bhandari S Ganguly I Agarwal P Munaganuru N Gupta N Singh A. Relationship of number of embryos transferred with perinatal outcome of singleton pregnancy. J Reprod Infertil. (2017) 18:179–84. PMC5359855 28377897

[18] Dai X Gao T Xia X Cao F Yu C Li T. Analysis of biochemical and clinical pregnancy loss between frozen-thawed embryo transfer of blastocysts and day 3 cleavage embryos in young women: A comprehensive comparison. Front Endocrinol (Lausanne). (2021) 12:785658. doi: 10.3389/fendo.2021.785658 35002968 PMC8740266

[19] De Sutter P. Single embryo transfer (set) not only leads to a reduction in twinning rates after IVF/ICSI, but also improves obstetrical and perinatal outcome of singletons. Verh K Acad Geneeskd Belg. (2006) 68:319–27. 17313092

[20] Luke B Brown MB Stern JE Grainger DA Klein N Cedars M. Effect of embryo transfer number on singleton and twin implantation pregnancy outcomes after assisted reproductive technology. J Reprod Med. (2010) 55:387–94. 21043364

[21] Shahbazi MN. Mechanisms of human embryo development: from cell fate to tissue shape and back. Development. (2020) 147. doi: 10.1242/dev.190629 PMC7375473 32680920

[22] Zhai J Xiao Z Wang Y Wang H. Human embryonic development: from peri-implantation to gastrulation. Trends Cell Biol. (2022) 32:18–29. doi: 10.1016/j.tcb.2021.07.008 34417090

[23] Niakan KK Han J Pedersen RA Simon C Pera RA. Human pre-implantation embryo development. Development. (2012) 139:829–41. doi: 10.1242/dev.060426 PMC3274351 22318624

[24] Peters BP Krzesicki RF Hartle RJ Perini F Ruddon RW. A kinetic comparison of the processing and secretion of the alpha beta dimer and the uncombined alpha and beta subunits of chorionic gonadotropin synthesized by human choriocarcinoma cells. J Biol Chem. (1984) 259:15123–30. doi: 10.1016/S0021-9258(17)42523-3 6210286

[25] Glasser SR Julian J Munir MI Soares MJ. Biological markers during early pregnancy: trophoblastic signals of the peri-implantation period. Environ Health Perspect. (1987) 74:129–47. doi: 10.1289/ehp.8774129 PMC1474497 3319548

[26] Rossant J Tam PPL. Early human embryonic development: Blastocyst formation to gastrulation. Dev Cell. (2022) 57:152–65. doi: 10.1016/j.devcel.2021.12.022 35077679

[27] Mercader A Valbuena D Simon C. Human embryo culture. Methods Enzymol. (2006) 420:3–18. doi: 10.1016/S0076-6879(06)20001-6 17161690

[28] Ellish NJ Saboda K O'Connor J Nasca PC Stanek EJ Boyle C. A prospective study of early pregnancy loss. Hum Reprod. (1996) 11:406–12. doi: 10.1093/HUMREP/11.2.406 8671233

[29] Macklon NS Geraedts JP Fauser BC. Conception to ongoing pregnancy: the 'black box' of early pregnancy loss. Hum Reprod Update. (2002) 8:333–43. doi: 10.1093/humupd/8.4.333 12206468

[30] Larsen EC Christiansen OB Kolte AM Macklon N. New insights into mechanisms behind miscarriage. BMC Med. (2013) 11:154. doi: 10.1186/1741-7015-11-154 23803387 PMC3699442

[31] Christiansen OB Nielsen HS Kolte AM. Inflammation and miscarriage. Semin Fetal Neonatal Med. (2006) 11:302–8. doi: 10.1016/j.siny.2006.03.001 16682265

[32] Vexo LE Stormlund S Landersoe SK Jorgensen HL Humaidan P Bergh C. Low-grade inflammation is negatively associated with live birth in women undergoing IVF. Reprod BioMed Online. (2023) 46:302–11. doi: 10.1016/j.rbmo.2022.10.004 36446681

[33] Zhao Y Man GCW Zhang R Wong CK Chen X Chung JP. A prospective study comparing the inflammation-related cytokine and chemokine profile from the day of blastocyst transfer to 7 weeks of gestation between pregnancies that did or did not result in a miscarriage. J Reprod Immunol. (2022) 154:103755. doi: 10.1016/j.jri.2022.103755 36272272

[34] Wong KY Tan HH Allen JC Chan J Ee TX Chua KH. Outcomes and cost analysis of single-embryo transfer versus double-embryo transfer. Womens Health (Lond). (2023) 19:17455057231206312. doi: 10.1177/17455057231206312 37899602 PMC10617257

[35] Mejia RB Capper EA Summers KM Ten Eyck P Van Voorhis BJ. Elective transfer of one embryo is associated with a higher cumulative live birth rate and improved perinatal outcomes compared to the transfer of two embryos with *in vitro* fertilization. F S Rep. (2021) 2:50–7. doi: 10.1016/j.xfre.2020.10.011 PMC8244291 34223273

[36] Clua E Tur R Coroleu B Boada M Rodriguez I Barri PN. Elective single-embryo transfer in oocyte donation programmes: Should it be the rule? Reprod BioMed Online. (2012) 25:642–8. doi: 10.1016/j.rbmo.2012.09.008 23069742

[37] Melado Vidales L Lawrenz B Vitorino RL Patel R Ruiz FJ Marques LM. Clinical and laboratory parameters associated with cycle outcomes in patients undergoing euploid frozen blastocyst transfer. Reprod BioMed Online. (2023) 46:917–25. doi: 10.1016/j.rbmo.2023.02.014 37062636

[38] Practice Committee of the American Society for Reproductive, M. and A.a.o. the Practice Committee for the Society for Assisted Reproductive Technologies. Guidance on the limits to the number of embryos to transfer: a committee opinion. Fertil Steril. (2021) 116:651–4. doi: 10.1016/j.fertnstert.2021.06.050 34330423

[39] Transfer EGGotNoEt Alteri A Arroyo G Baccino G Craciunas L De Geyter C. ESHRE guideline: number of embryos to transfer during IVF/ICSIdagger. Hum Reprod. (2024) 39:647–57. doi: 10.1093/humrep/deae010 PMC10988112 38364208

